# NtbHLH1, a JAF13-like bHLH, interacts with NtMYB6 to enhance proanthocyanidin accumulation in Chinese Narcissus

**DOI:** 10.1186/s12870-021-03050-1

**Published:** 2021-06-16

**Authors:** Yuxin Fan, Jiayu Peng, Jiacheng Wu, Ping Zhou, Ruijie He, Andrew C. Allan, Lihui Zeng

**Affiliations:** 1 Institute of Genetics and Breeding in Horticultural Plants, Fujian Agriculture and Forestry University, Fuzhou, 350002 China; 2 College of Horticulture, Fujian Agriculture and Forestry University, Fuzhou, 350002 China; 3 The New Zealand Institute for Plant & Food Research, Mt Albert Research Centre, Private Bag 92169, Auckland, New Zealand; 4 School of Biological Sciences, University of Auckland, Private Bag 92019, Auckland, 1142 New Zealand

**Keywords:** Chinese Narcissus, Flavonoid, Proanthocyanidin, BHLH, MYB

## Abstract

**Background:**

Flavonoid biosynthesis in plants is primarily regulated at the transcriptional level by transcription factors modulating the expression of genes encoding enzymes in the flavonoid pathway. One of the most studied transcription factor complexes involved in this regulation consists of a MYB, bHLH and WD40. However, in Chinese Narcissus (*Narcissus tazetta* L. var. *chinensis*), a popular monocot bulb flower, the regulatory mechanism of flavonoid biosynthesis remains unclear.

**Results:**

In this work, genes related to the regulatory complex, *NtbHLH1* and a R2R3-MYB *NtMYB6,* were cloned from Chinese Narcissus. Phylogenetic analysis indicated that *NtbHLH1* belongs to the JAF13 clade of bHLH IIIf subgroup, while *NtMYB6* was highly homologous to positive regulators of proanthocyanidin biosynthesis. Both *NtbHLH1* and *NtMYB6* have highest expression levels in basal plates of Narcissus, where there is an accumulation of proanthocyanidin. Ectopic over expression of *NtbHLH1* in tobacco resulted in an increase in anthocyanin accumulation in flowers, and an up-regulation of expression of the endogenous tobacco bHLH *AN1* and flavonoid biosynthesis genes. In contrast, the expression level of *LAR* gene was significantly increased in *NtMYB6*-transgenic tobacco. Dual luciferase assays showed that co-infiltration of *NtbHLH1* and *NtMYB6* significantly activated the promoter of Chinese Narcissus *DFR* gene. Furthermore, a yeast two-hybrid assay confirmed that NtbHLH1 interacts with NtMYB6.

**Conclusions:**

Our results suggest that NtbHLH1 may function as a regulatory partner by interacting directly with NtMYB6 to enhance proanthocyanidin accumulation in Chinese Narcissus.

**Supplementary Information:**

The online version contains supplementary material available at 10.1186/s12870-021-03050-1.

## Background

Flavonoids, including anthocyanins and proanthocyanidins (PAs), are major metabolites often pigmented and are abundant in the seed coats, leaves, fruits, flowers, and bark of many plant species [9]. Expression of genes encoding enzymes of flavonoid-specific biosynthesis is regulated by a conserved transcription factor complex, which is composed of R2R3-MYB, bHLH, and WD40 components, termed the MBW complex.

The bHLH transcription factor family is the second largest transcription factor family found in plants, which are distinguished by containing two functional regions in the protein. Approximately 200 amino acids at the N-terminus of the protein sequence is involved in interaction with the MYB transcription factor. The next 200 amino acids bind to a WD40 protein and may also contact with RNA polymerase II to produce transcriptional activation [12]. The remaining C-terminal bHLH domain can form homodimers or heterodimers together with other bHLH proteins [16]. BHLH transcription factors are reported to have roles in light signal transduction [34], formation of root hairs [25], transduction of gibberellin signals [21] and synthesis of flavonoids and anthocyanins [24].

The bHLH family is divided into 26 sub-families [30], amongst this the IIIf subfamily is proposed to regulate flavonoid biosynthesis [15]. *Booster1* (B) and *Red1* (R) are the first flavonoid-related bHLH transcription factors as identified in maize [8]. Other bHLHs related to flavonoid biosynthesis were subsequently studied, such as *PhAN1* and *PhJAF13* in petunia [33, 35], *AtTT8* and *AtGLABRA3* in *Arabidopsis* [14, 27], and *VvMYC1* and *VvMYCA1* in grapes [17].

The IIIf subfamily bHLHs can be further divided into two clades: JAF13 clade and AN1 clade. In Solanaceous plants, AN1 is directly involved in the activation of the biosynthetic genes, whereas JAF13 is involved in the regulation of AN1 transcription [26]. In maize, overexpression of *ZmLC* can promote the expression of *ZmDFR* and *ZmANS*, key genes of the flavonoid biosynthesis pathway, and promote the accumulation of anthocyanins [20]. Therefore, in different species, and in particular across the dicot and monocot division, the function of bHLHs may differ.

R2R3-MYB proteins are the largest class of transcription factors in plants and are involved in a wide range of regulatory functions including regulation of various secondary metabolites [10]. R2R3-MYB transcription factors have highly conserved DNA-MYB domains, which consist of a series of highly conserved amino acid sequences and spacer sequences [23].

Zea mays *ZmC1* was the first identified R2R3-MYB transcription factor shown to regulate anthocyanin biosynthesis [29]. Most MYBs found in plants that can promote flavonoid metabolism are R2R3-MYBs. For example, *PyMYB10* regulates anthocyanin biosynthesis in pears [13],*DkMYB4* in persimmons promotes the accumulation of proanthocyanidins by directly binding promoters of some structural genes of the proanthocyanidin pathway [1, 2]. *VvMYBPA1* activates *VvLAR* and *VvANR* promoters to up-regulate proanthocyanidin biosynthesis in grape peel [7].

The MYB-bHLH-WD40 (MBW) complex has a crucial role in the regulation of flavonoid biosynthesis. In heterologous systems, the monocot members of this complex can activate anthocyanin expression in dicots; petunia transformed with *ZmC1* has activated expression of *PhCHSJ* and *PhDFRA* [32]. Tobacco transformed with *ZmLC* can activate the expression of tobacco *CHS* and *DFR* [28]. Freesia FhGL3L and FhTT8L can interact with AtPAP1 and AtTT2, thereby regulate the flavonoid biosynthesis pathway [22].

Chinese narcissus (*Narcissus tazetta* L. var. *chinensis*) which belongs to the monocot Amaryllidaceae family is a perennial bulbous plant and shows high ornamental value [39]. Flower colors of Chinese narcissus are only yellow or white. The composition of flavonoid compounds in petals and corona was flavonols, but no anthocyanins are found. In addition, proanthocyanidins are found accumulate mainly in basal plates, but not detected in flowers [36]. Our recent research found that *NtMYB2*, *NtMYB3* and *NtMYB5* [38] are transcriptional repressors of flavonoid biosynthesis in Chinese narcissus. However, activation of flavonoid biosynthesis in Chinese narcissus is still not clear [4, 5].

In the current investigation, a JAF13-like bHLH homolog, *NtbHLH1* and a R2R3-MYB homolog, *NtMYB6* were isolated from Chinese narcissus and functionally characterized. *NtbHLH1* and *NtMYB6* were ectopically expressed in transgenic tobacco, increasing the expression level of flavonoid and proanthocyanidin biosynthesis genes. Dual luciferase assays showed that co-expression of *NtbHLH1* and *NtMYB6* significantly activated the promoter of Chinese narcissus *DFR* gene (*NtDFR*). Yeast two-hybrid (Y2H) analysis confirmed the interaction of *NtbHLH1* and *NtMYB6.* Our results suggest that *NtbHLH1* plays a role in flavonoid biosynthesis and interacts with *NtMYB6* to directly promote proanthocyanidin biosynthesis in Chinese narcissus.

## Results

### Cloning and Sequence Analysis of *NtbHLH1*

**Fig. 1:**
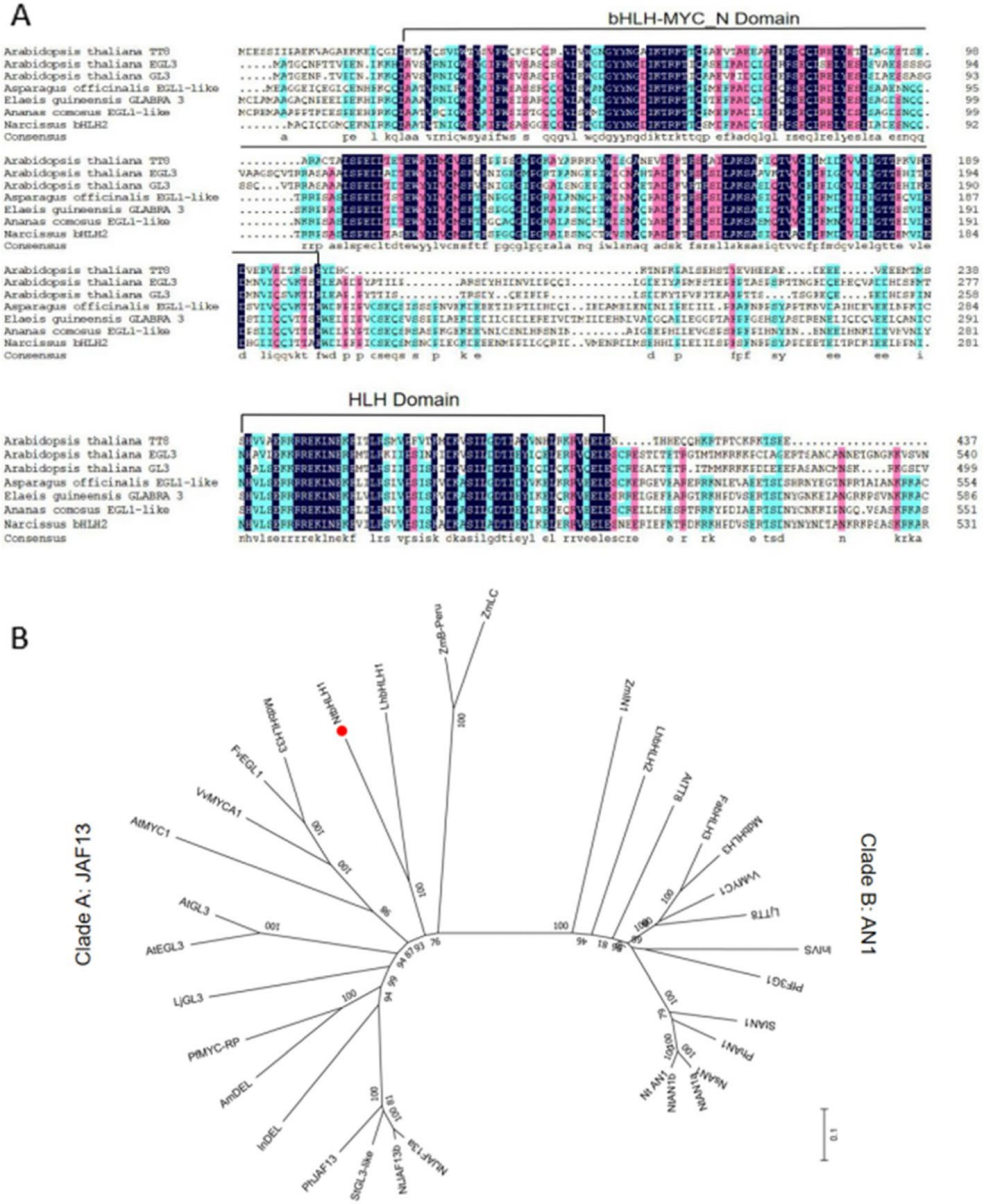
(**A**) Amino acid sequence comparison between NtbHLH1 and six flavonoid-related bHLHs. Two conserved domains (bHLH-MYC_N domain and HLH domain) among known bHLH TFs regulating anthocyanin flavonoid biosynthesis are underlined. (**B**) A neighbor-joining phylogenetic tree of IIIf subfamily plant bHLHs. Numbers next to the nodes are bootstrap values from 1,000 replications. The tree is drawn to scale, with branch lengths in the same units as those of the evolutionary distances that were used to infer the phylogenetic tree. The following deduced amino acid sequences were retrieved from GenBank databases: *Arabidopsis thaliana* AtTT8 (NP_192720.2), AtEGL3 (NP_001332705.1), AtGL3 (NP_001185302.1); *Asparagus officinalis* EGL1-like (XP_020260845.1); *Elaeis guineensis GLABRA 3* (XP_010918535.1); *Ananas comosus* EGL1-like (XP_020084606.1); Chinese narcissus *NtbHLH1* (QDS02912.1). Zea mays *ZmLC* (P13526.1), ZmB-Peru (CAA40544.1), *ZmIN1* (AAB03841.1); *Lilium hybrid division I LhbHLH1*(BAE20057.1), *LhbHLH2*(BAE20058.1); Chinese narcissus NtbHLH1 (QDS02912.1); Malus domestica MdbHLH33 (ABB84474.1), MdbHLH3 (ADL36597.1); *Fragaria vesca subsp. vesca* FvEGL1 (XP_004308377.1); *Vitis vinifera* VvMYC1 (NP_001268182.1), *VvMYCA1* (NP_001267954.1); *Arabidopsis thaliana* AtMYC (NP_191957.2), *Lotus japonicus* LjGL3 (BAJ10680.1), LjTT8 (BAH28881.1); *Perilla frutescens* PfMYC-RP (BAA75513.1), PfF3G1 (BAC56998.1); *Antirrhinum majus* AmDEL (AAA32663.1); *Ipomoea ni*l InDEL (XP_019171149.1), InIVS (XP_019197480.1); *Petunia x hybrida* phJAF13 (AAC39455.1), PhAN1 (AAG25927.1); *Solanum tuberosum* StGL3-like (NP_001275132.1), StAN1 (ALA13578.1); *Nicotiana tabacum* NtAN1a (NP_001312042.1), NtAN1b (NP_001312452.1), NtJAF13a (NP_001311775.1), NtJAF13b (AHY00341.1); *Fragaria x ananassa FabHLH3* (AFL02463.1); *Nicotiana sylvestris* NsAN1 (NP_001289495.1); *Nicotiana tomentosiformis* NtAN1 (NP_001289454.1)

Previously, an RNA-Seq-based transcriptome database from flowers and basal plates of Chinese Narcissus was generated [36]. By blast-match to sequences in this database, one potential flavonoid-related bHLH gene was identified and designated as *NtbHLH1*. The 3´ end sequence of *NtbHLH1* was obtained by 3´ RACE and then the full length of open reading frame (ORF) was cloned by RT-PCR (reverse transcription PCR) (Supplemental Fig. 1) (GenBank number QDS02912.1). *NtbHLH1* has an ORF of 1,896 bp, encoding a polypeptide of 632 amino acids. The predicted NtbHLH1 protein contains two domains, N-terminal bHLH-MYC_N domain and C-terminal HLH domain that are conserved in flavonoid-related bHLH-type TFs (Fig. 1A). These two domains have been predicted to be important for the bHLH protein to perform transcriptional regulatory function [37].

Phylogenetic tree analysis showed that flavonoid-related bHLHs which belong to subgroup IIIf were clearly classified into two clades, JAF13-like clade and AN1-like clade (Fig. 1B). JAF13-like clade included AtEGL3, AtGL3 (*Arabidopsis thaliana*)*,* PhJAF13 (*Petunia x hybrid*) and LhbHLH1 (*Lilium hybrid division I*), whereas AN1-like clade contained *AtTT8* (*Arabidopsis thaliana*), *NtAN1a*, *NtAN1b* (*Nicotiana tabacum*) and *LhbHLH2* (*Lilium hybrid division I*). *NtbHLH1* was grouped into clade A, close to *LhbHLH1*.

### Expression pattern of *NtbHLH1* in Chinese narcissus

**Fig. 2:**
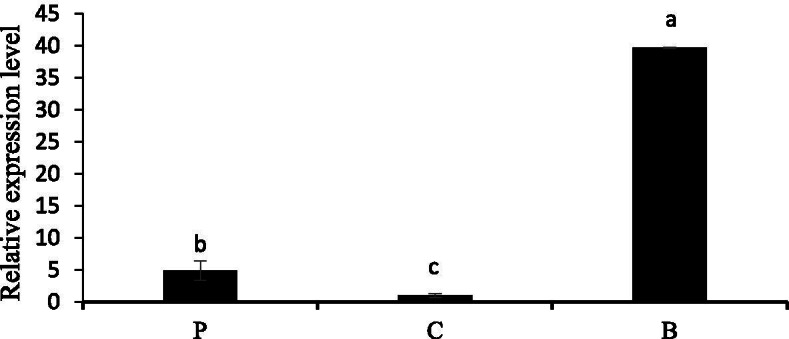
Expression of *NtbHLH1* in different tissues of Chinese narcissus. P indicates the petals. C denotes for corona. B represents for basal plates. The bars indicate the standard error of three biological replicates. Letter represents a significant difference at the level of *P* < 0.05 using SPSS statistical analysis

The expression pattern of *NtbHLH1* in different tissues of Chinese narcissus was analyzed by qRT-PCR (Quantitative real-time PCR) (Fig. 2). The expression level of *NtbHLH1* in basal plates was the highest, as compared to petals, and corona. This tissue has been shown to accumulate proanthocyanidin in previous research [36]. This result suggests that *NtbHLH1* has the potential to play a role in the biosynthesis of flavonoids in basal plates.

### Over-expression of *NtbHLH1* in Tobacco

**Fig. 3:**
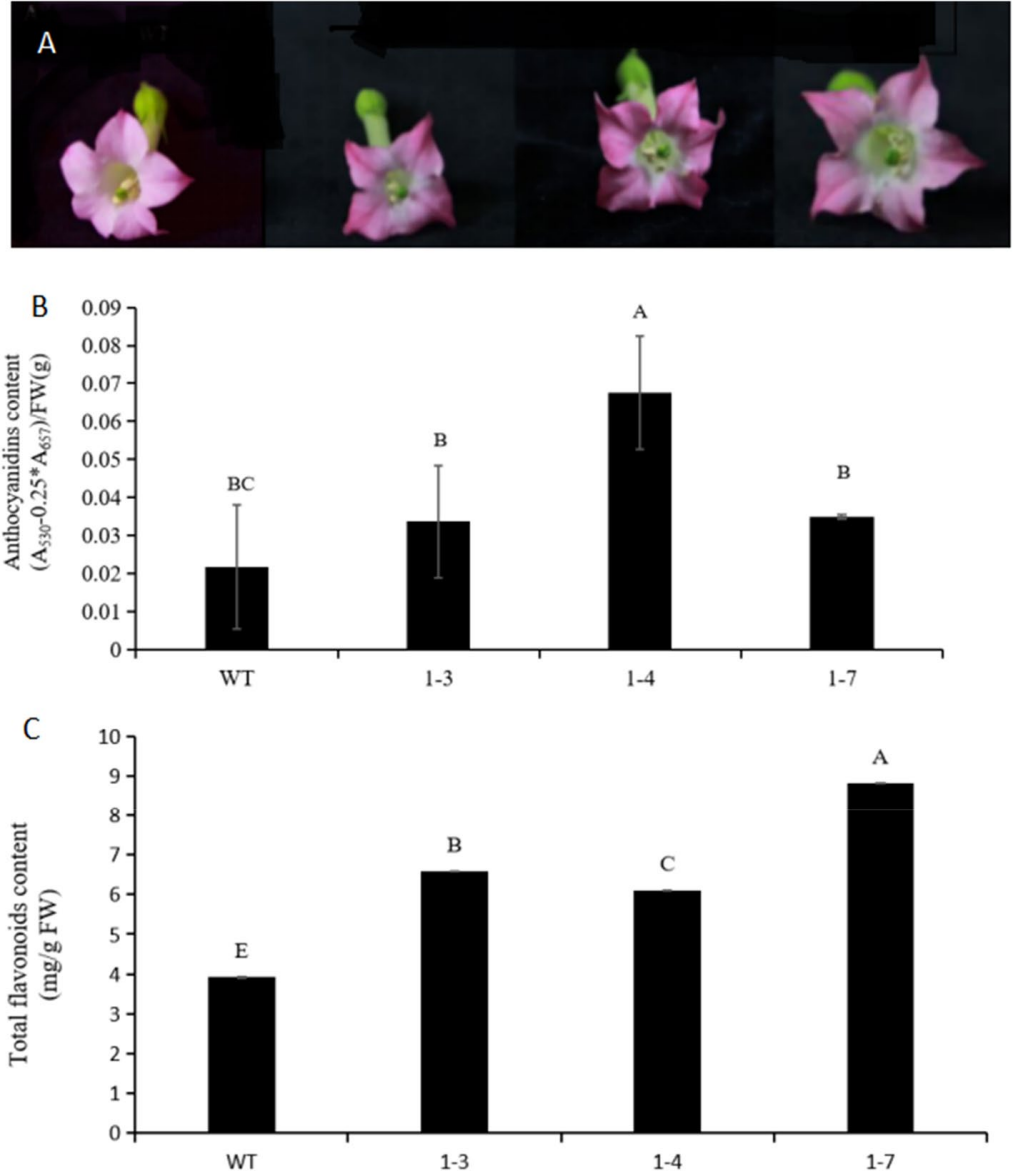
Anthocyanin contents of transgenic tobacco plants carrying *NtbHLH1* gene. (**A**) Flower color comparison between wild type (WT) and transgenic lines (1–3, 1–4 and 1–7). (**B**) Anthocyanin contents. (**C**) Total flavonoid contents. The bars represent the standard error of thrice biological replication. Small letters showed a significant difference at the level of *p* < 0.05 by using SPSS statistical analysis

Transgenic tobacco over-expressing *NtbHLH1* displayed an increase in red appearance in the petals as compared to controls (Fig. 3A). All flowers of transgenic lines had higher anthocyanin content than wild type tobacco. Floral anthocyanin content of three transgenic lines was between 1.7 to 3.24 fold higher than control tobacco petals (Fig. 3B). Total flavonoid content of the flowers of the three transgenic lines increased significantly compared to the control (Fig. 3C).

**Fig. 4:**
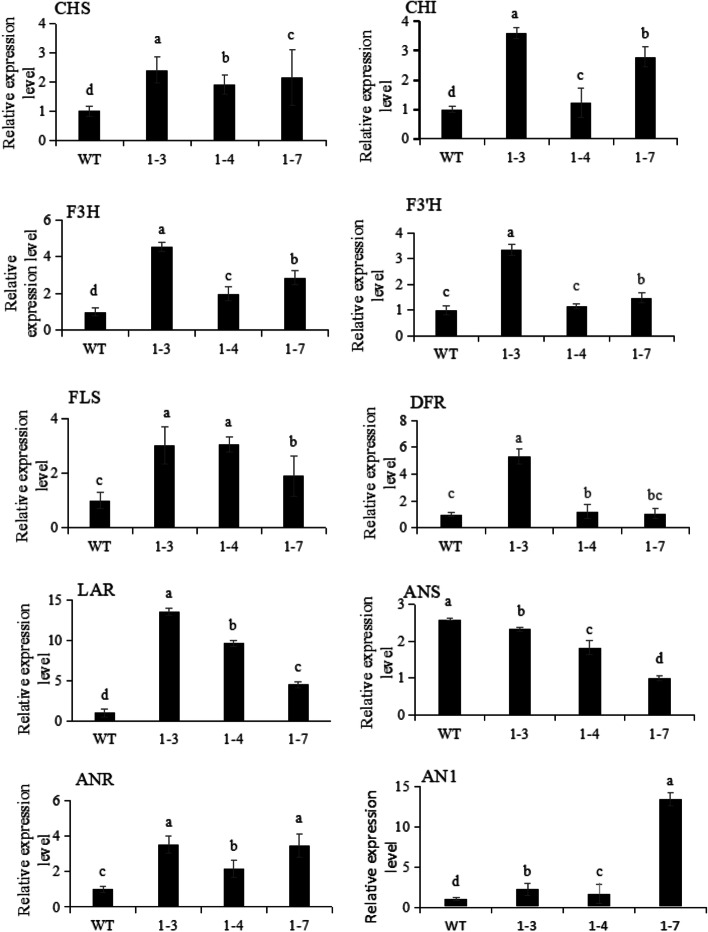
Expression analysis of flavonoid biosynthesis pathway genes in flowers of *NtbHLH1* transgenic tobacco. 1–3, 1–4 and 1–7 means three transgenic lines. The bars indicate the standard error of three biological replicates. Star represents the significant difference from wild type at the level of *p* < 0.05 using SPSS test

To check the effects of ectopic over-expression of *NtbHLH1* on endogenous tobacco genes encoding the tobacco flavonoid biosynthesis pathway, qRT-PCR analysis on the flowers was performed. The results indicated that over-expression of *NtbHLH1* affected the transcript levels of tobacco flavonoid biosynthesis genes. Expression of the tobacco bHLH *AN1* and the structural genes *CHS*, *FLS*, *LAR*, *F3H*, *DFR*, *ANR* were up-regulated (Fig. 4). The transcript level of early pathway gene *CHS* was 1.3 to 2.3 times higher and late pathway gene *LAR* was 4.5 to 13.5 times higher in the transgenic lines than the control. These data indicate that *NtbHLH1* promotes not only anthocyanin biosynthesis in the petals but also flavonoid biosynthesis in transgenic tobacco plants.

### Cloning and sequence analysis of *NtMYB6*

**Fig. 5:**
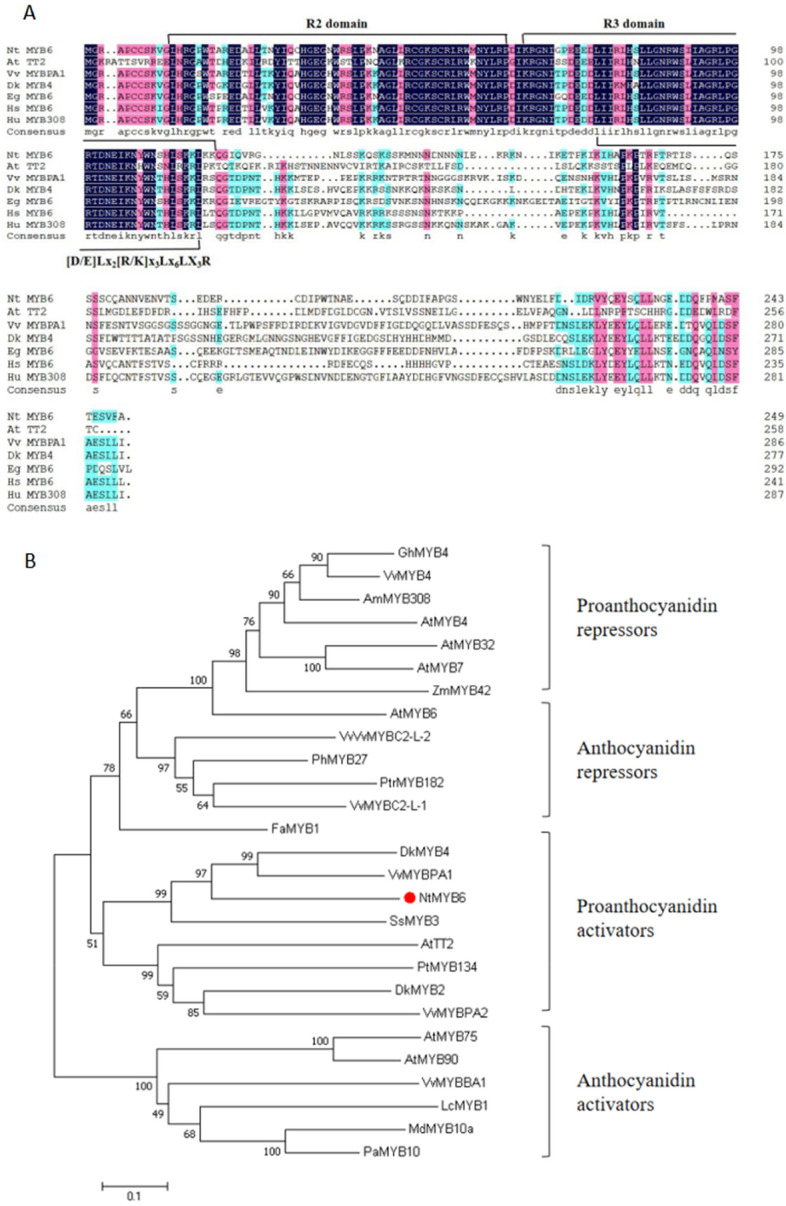
(**A**) Comparison of amino acid sequences between *NtMYB6* and nine R2R3- MYBs. Three conserved domains (R2 domain, R3 domain and [D/E]Lx_2_[R/K]x_3_Lx_6_LX_3_R) are underlined. (**B**) A neighbor-joining phylogenetic tree of plant R2R3-MYB sequences. Numbers next to the nodes are bootstrap values from 1,000 replications. The tree is drawn to scale, with branch lengths in the same units as those of the evolutionary distances that were used to infer the phylogenetic tree. The following deduced amino acid sequences were retrieved from GenBank databases: Chinese narcissus NtMYB6 (KY645961.1); *Gossypium hirsutum* GhMYB4 (XP_016708004.1); *Antirrhinum majus* AmMYB308 (P81393.1); *Arabidopsis thaliana AtMYB4* (NP_195574.1),AtMYB7 (NM_127224.6), AtMYB32 (NP_195225.1), AtMYB6 (NP_192684.1), AtTT2 (NP_198405.1), AtMYB75 (NM_104541.4), AtMYB90 (NM_105310.4); *Zea mays* ZmMYB42 (HQ858694.1); *Vitis vinifera* VvMYBC2-L-1 (NP_001268133.1), VvMYBC2-L2 (GQ903730.1), VvMYB4 (NP_001268129.1), VvMYBPA1 (NP_001268160.1), VvMYBPA2 (ACK56131.1), VvMYBBA1 (XP_010664911.1); *Fragaria x ananassa* FaMYB1 (AF401220.1); *Petunia x hybrida* PhMYB27(AHX24372.1); *Populus tremula x Populus tremuloides* PtrMYB182 (AJI76863.1); *Diospyros kaki* DkMYB2 (BAI49719.1), DkMYB4 (BAI49721.1), *Solenostemon scutellarioides* SsMYB3 (EF522163.1); *Populus tremuloides* PtMYB134 (ACR83705.1); *Litchi chinensis* LcMYB1 (KY302802.1); *Malus x domestica* MdMYB10a (DQ267898.1); *Prunus avium L. Tieton* PaMYB10 (KR259845.1), *Hibiscus syriacus* HsMYB6 (KAE8700978.1); *Herrania umbratica* HuMYB308 (XP_021280377.1); (BAI49721.1); *Elaeis guineensis* EgMYB6 (XP_019705406.1)

Because *NtbHLH1* is mainly expressed in basal plates of Narcissus, we analysed the RNA-Seq database [36] for potential transcriptional partners for this bHLH. A R2R3-MYB was identified which also showed higher expression levels in basal plates. This R2R3-MYB gene was cloned (Supplemental Fig. 3) and named *NtMYB6*. Sequence analysis showed that *NtMYB6* has a full length ORF of 768 bp encoding a polypeptide of 256 amino acids (GenBank number KY645961). Protein sequence alignment between *NtMYB6* and other R2R3-MYBs showed that *NtMYB6* contained the conserved R2 and R3 DNA binding domains at the N-terminus (Fig. 5A).

Phylogenetic analysis showed that *NtMYB6* was placed in a clade of R2R3-MYB proteins that include DkMYB4, VvMYBPA1 and AtTT2, which have been characterized as regulatory activators of proanthocyanidin biosynthesis related gene expression, suggesting that *NtMYB6* may act as a positive regulator of Narcissus proanthocyanidin biosynthesis (Fig. 5B).

**Fig. 6:**
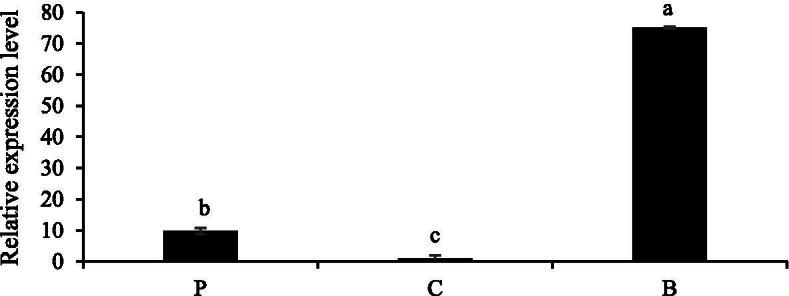
Expression of *NtMYB6* in different tissues of Chinese narcissus. P indicates the petals. C denotes for corona. B represents for basal plates. The bars indicate the standard error of three biological replicates. Letter represents a significant difference at the level of *P* < 0.05 using SPSS statistical analysis

The expression of *NtMYB6* gene in Chinese narcissus was examined by real-time qRT-PCR. The results confirmed that *NtMYB6* expression was higher in basal plates. Expression levels of *NtMYB6* in perianth and corona were significantly lower (Fig. 6).

### Over-Expression of *NtMYB6* in tobacco

**Fig. 7:**
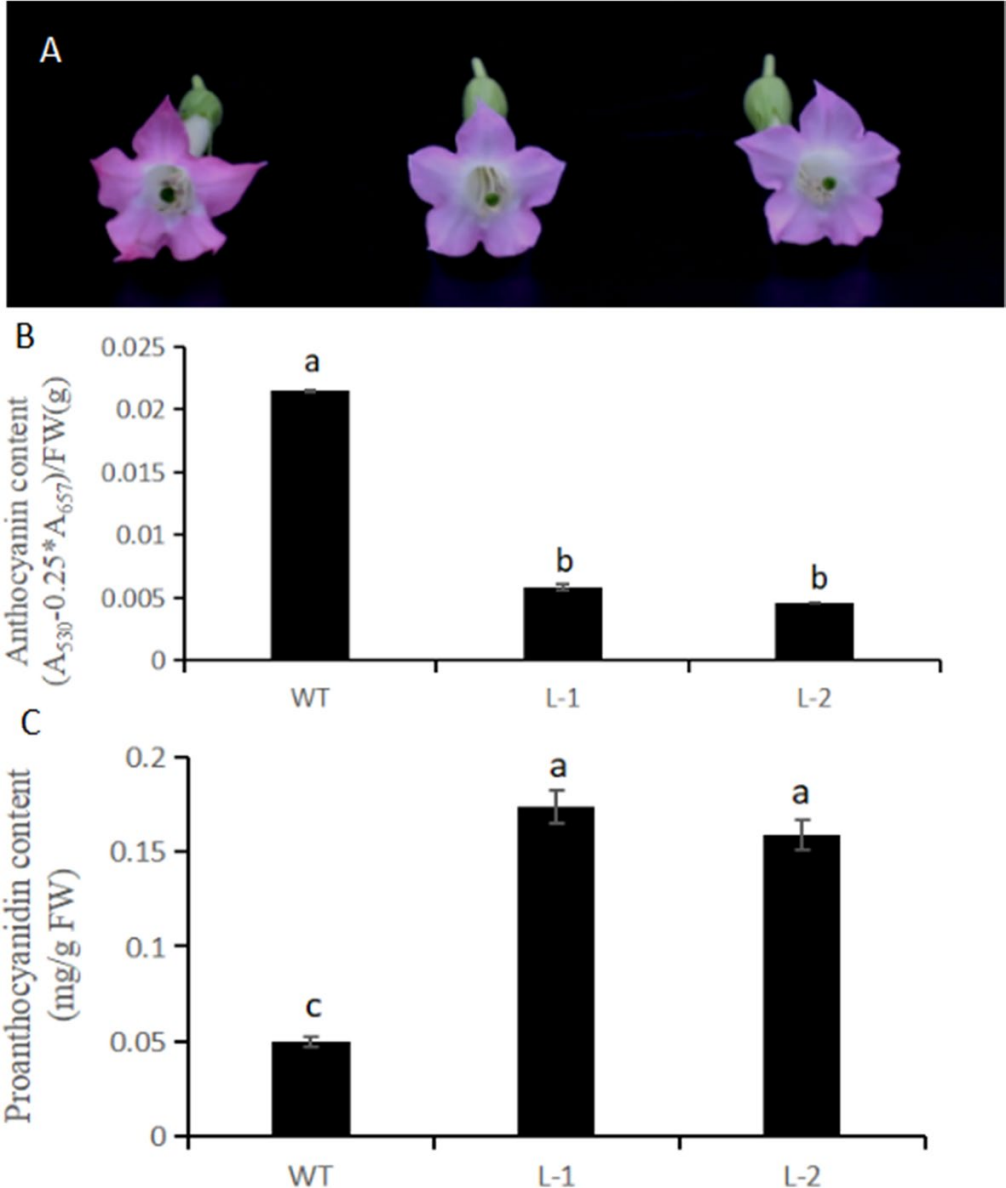
Transgenic tobacco plants carrying *NtMYB6* gene. (**A**) Flower color of wild type (WT) and transgenic lines (L-1 and L-2). (**B**) Anthocyanin contents. (**C**) Proanthocyanidin contents. The bars represent the standard error of thrice biological replication. Small letters showed a significant difference at the level of *p* < 0.05 by using SPSS statistical analysis

**Fig. 8:**
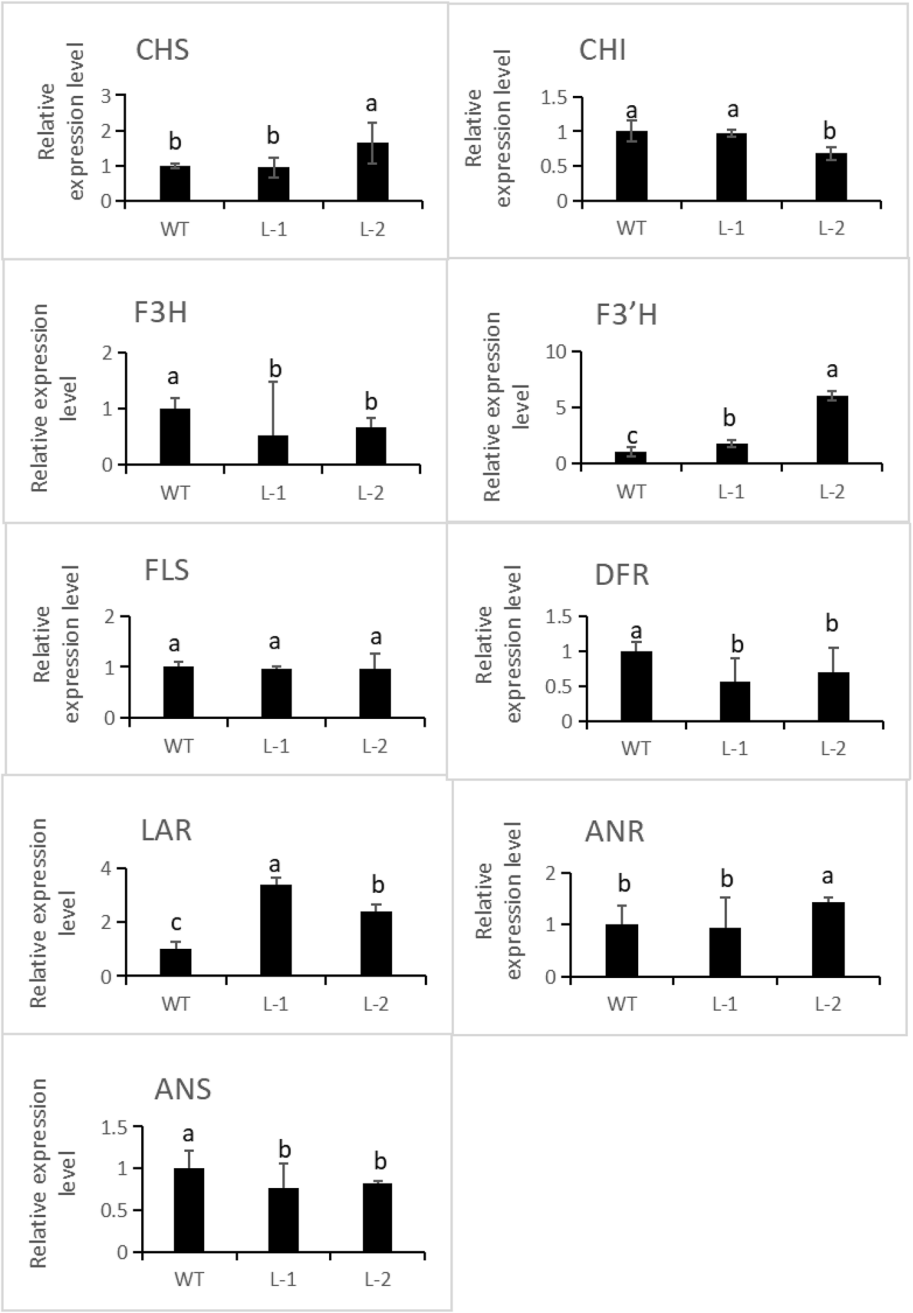
Expression analysis of flavonoid biosynthesis pathway genes in flowers of *NtbMYB6* transgenic tobacco. L-1 and L-2 means the transgenic line. The bars indicate the standard error of three biological replicates. Star represents the significant difference from wild type at the level of *p* < 0.05 using SPSS test

Compared to the wild type tobacco, the flowers of tobacco expressing the *NtMYB6* gene displayed a decrease in color (Fig. 7A). Anthocyanin content decreased and proanthocyanidin contents increased significantly in transgenic flowers (Fig. 7B, C). QRT-PCR analysis showed that expression levels of tobacco F3’H and LAR were significantly up-regulated, while the expression levels of other flavonoid pathway genes was either unaffected or down-regulated (Fig. 8).

### Dual luciferase assay

**Fig. 9:**
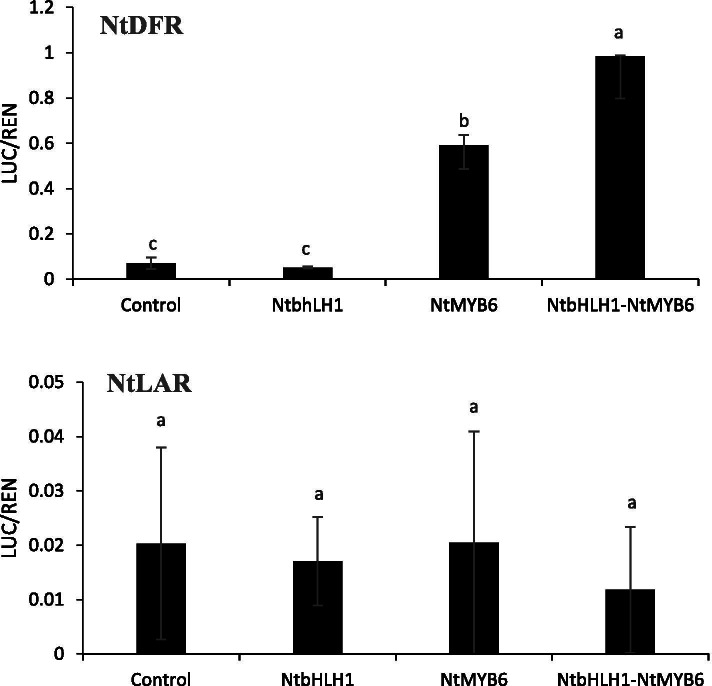
Dual luciferase assay of Narcissus *DFR* or *LAR* promoter activity. Luc and Ren values were measured 3 days after infiltration. The bars represent the standard error of thrice biological replication. Small letters showed a significant difference at the level of *p* < 0.05 by using SPSS statistical analysis

A dual luciferase system in *Agrobacterium tumefaciens*-infiltrated *Nicotiana benthamiana* leaves was used to investigate the function of *NtbHLH1* and *NtMYB6*. *NtbHLH1* and *NtMYB6* were independently infiltrated or co-infiltrated into tobacco leaves, along with the promoter of Narcissus *DFR* or *LAR* fused to luciferase. It was found that *NtbHLH1* alone did not activate the *NtDFR* promoter, however, *NtMYB6* alone can activate the *NtDFR* promoter (Fig. 9). Co-infiltration of both *NtbHLH1* and *NtMYB6* showed a significant increase in *NtDFR* promoter activity, resulting in approximately 14-fold increase in the promoter activity of *NtDFR* compared to the control and eightfold increase compared to *NtMYB6* alone. *NtbHLH1* and *NtMYB6* alone or co-infiltration showed little effect on *NtLAR* promoter (Fig. 9). These results indicate that *NtMYB6* mediated activation of *NtDFR* promoter is enhanced by the bHLH TF *NtbHLH1*.

### Protein–Protein Interaction between NtbHLH1 and NtMYB6

**Fig. 10:**
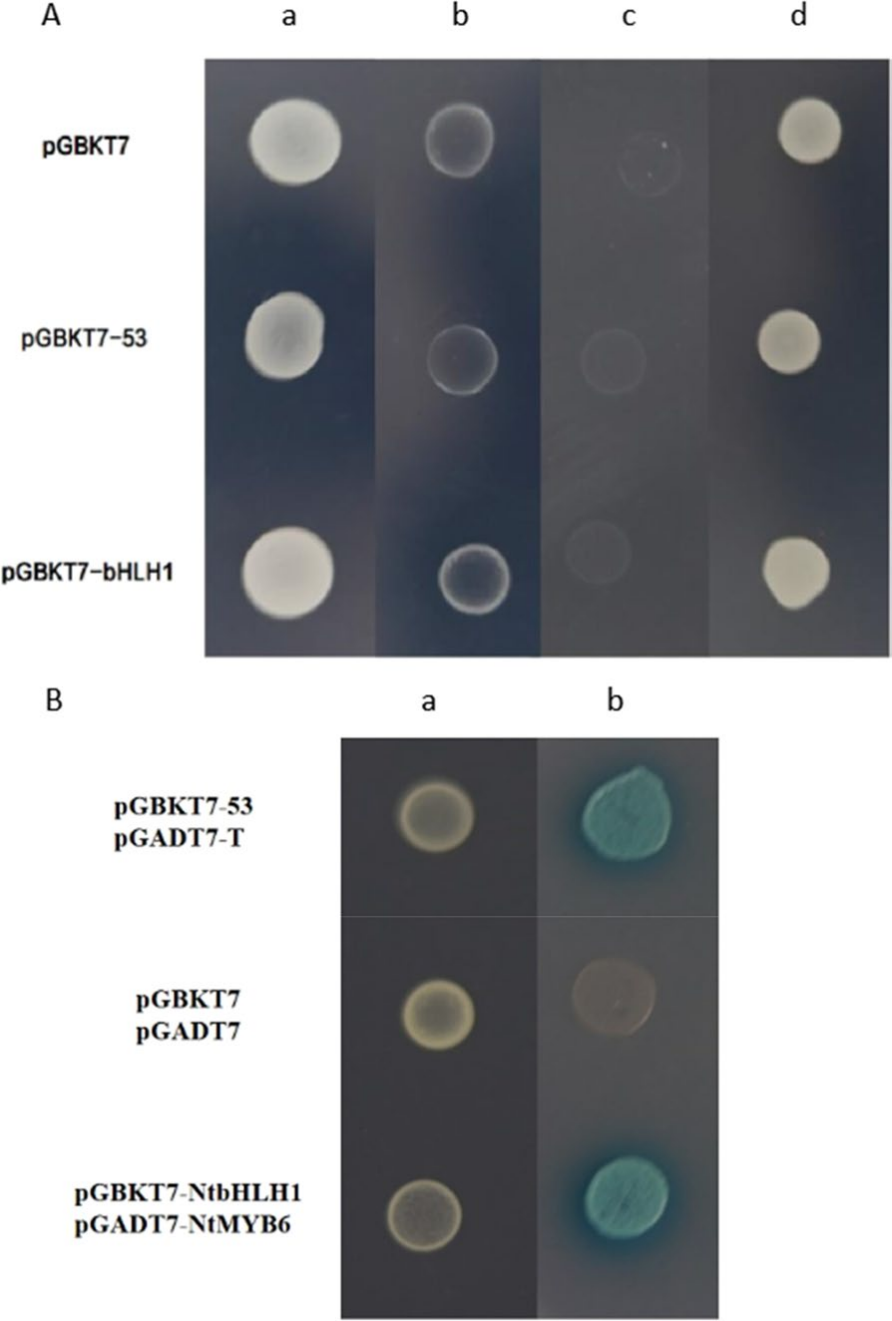
(**A**) Auto-activation of the bait plasmid. a, b, c and d indicate the different mediums of SD/-Trp, SD/-Trp/-Ade, SD/-Trp/-His and SD/-Trp/X-α-Gal. (**B**) Yeast two-hybrid analysis of interactions between *NtbHLH1* and *NtMYB6*. a, b indicate the different mediums of SD/-Trp/-Leu, SD/-Trp/-Leu/-Ade/-His/X-α-Gal

Yeast two-hybrid (Y2H) was performed to investigate the interaction between *NtbHLH1* and *NtMYB6*. Firstly, the auto-activation was tested for *NtbHLH1*. Auto-activation test results showed that pGBKT7-NtbHLH1 was not able to grow in SD/-Trp/-Ade and SD/-Trp/-His medium, and did not turn blue in SD/-Trp /X-ɑ-Gal medium. This indicates that NtbHLH1 does not have auto-activation activity (Fig. 10A). pGADT7 and pGBKT7 empty vectors were used as negative controls. Yeast cells transformed with BD-NtbHLH1 and AD-NtMYB6 showed growth on both SD/-Trp/-Leu and SD/-Trp/-Leu/-Ade/-His media, and their color turned to blue when grown on SD/-Trp/-Leu/-His/-Ade /X-ɑ-Gal medium. By contrast, the negative controls could grow on SD/-Trp/-Leu medium, but not on SD/-Trp/-Leu/-Ade/-His medium (Fig. 10B). These results confirm the protein–protein interaction between NtbHLH1 and NtMYB6.

## Discussion

### NtbHLLH1 is a bHLH Transcription Factor That Regulates Flavonoid Biosynthesis

In this study, we isolated a bHLH transcription factor *NtbHLH1* from Chinese Narcissus. NtbHLH1 has a conserved bHLH-MYC_N terminal structure and a HLH domain, and belongs to the JAF13 clade of IIIf subfamily which includes PhJAF13 (*Petunia hybrida*), ZmLC (*maize*), and AtGL3 (*Arabidopsis*). In dicotyledons, JAF13-like bHLH interacts with R2R3-MYB protein promoting the expression of a AN1-like bHLH, and then AN1 interacts with an R2R3-MYB to activate the expression of biosynthetic genes in the flavonoid pathway [3]. In Arabidopsis, JAF13-like *AtGL3* is expressed at an early stage of anthocyanin synthesis, and an MBW complex PAP1-GL3-TTG1 was first formed. This MBW complex activates the expression of *AtTT8*, and then another MBW complex which includes AtTT8 activates the expression of structural genes related to flavonoid biosynthesis [6].

Ectopic expression of *NtbHLH1* in tobacco demonstrates that it functions as a positive regulator of flavonoid biosynthesis. The expression of *NtbHLH1* increased anthocyanin and total flavonoid contents of transgenic tobacco petals by activating the expression of tobacco genes encoding anthocyanin-related enzymes and the bHLH *AN1*, suggesting *NtbHLH1* may regulate both *AN1* and structural genes directly. In monocotyledons, JAF13-like bHLHs can interact with MYBs to directly promote the expression of structural genes in the flavonoid biosynthesis pathway. For example, *Freesia hybrida* FhGL3 and FhTT8 can activate transcription of *AtDFR* with AtAPA1 and the activation capacity of FhGL3 with AtPAP1 is higher than FhTT8 with AtPAP1 [20]. Our results suggest that *NtbHLH1* may have a function similar to JAF13-like bHLHs in monocotyledons.

### NtMYB6 is an activator of proanthocyanidin biosynthesis

Phylogenetic analysis shows that NtMYB6 is highly similar to the R2R3-MYB transcription factors DkMYB4 and VvMYBPA1 which have been shown to activate proanthocyanidin biosynthesis. The ectopic overexpression of *VvMYBPA1* in *Arabidopsis* induced elevated biosynthesis of proanthocyanidins in roots, hypocotyls, and apical meristems. Transient expression assays revealed the ability of *VvMYBPA1* to activate the proanthocyanidin-specific branch point genes *VvANR* and *VvLAR1* [7]. Ectopic expression of *DkMYB4* induced proanthocyanidin accumulation in the kiwifruit callus and directly activates *DkANR* transcription [1, 2]. Ectopic expression of *NtMYB6* in tobacco activated the expression of *LAR* gene and induced PA accumulation in transgenic flowers suggesting *NtMYB6* possibly has similar regulatory functions in proanthocyanidin biosynthesis.

### NtbHLH1 interacts with NtMYB6 to activate *NtDFR* expression and promote proanthocyanidin biosynthesis

*NtbHLH1*, a flavonoid-related bHLH regulator, was found to have higher expression levels in basal plates compared to petals and corona of Chinese Narcissus. In the same tissue, the expression level of *NtMYB6* was also elevated. Previous studies have shown that proanthocyanidins were accumulated mainly in basal plates of Chinese Narcissus [36]. Therefore the expression profiles of *NtbHLH1* and *NtMYB6* were positively associated with the pattern of proanthocyanidin accumulation. Yeast two-hybrid experiment confirmed the interaction of NtbHLH1 with NtMYB6. The accumulation of proanthocyanidin is likely because the expression of *NtDFR* and *NtLAR* was significantly up-regulated in basal plates [36]. Dual luciferase reporter assays showed that combined with *NtbHLH1*, *NtMYB6* activated the expression of *NtDFR* more significantly, but in this heterologous system this combination did not activate the expression of *NtLAR*. However, ectopic expression of *NtMYB6* in tobacco can activate the expression of tobacco *LAR g*ene. Other factors may be involved in how *NtbHLH1* interacts with *NtMYB6* to promote activation of the Narcissus *LAR* gene. One of possible reasons why *NtDFR* has low expression level in petals and corona is the low expression levels of *NtbHLH1* and *NtMYB6*.

Our results are consistent with previous work that show bHLH and MYB proteins have essential roles in proanthocyanidin biosynthesis. Although NtbHLH1 is a JAF-13 like bHLH (Quattrocchio et al., 1998b; D'Amelia et al., 2014), it can form the MBW complex with R2R3-MYB to regulate structural genes of flavonoid biosynthesis directly in Chinese narcissus such as is seen in other monocotyledons. Only one flavonoid-related bHLH has been characterised in Chinese narcissus. Whether *NtbHLH1* interacts with other R2R3-MYBs to activate the flavonol biosynthesis in flowers needs further study.

## Conclusions

We have demonstrated that *NtbHLH1* functions as a positive regulator of flavonoid biosynthesis and *NtMYB6* positively regulates the proanthocyanidin biosynthesis. NtbHLH1 is likely to form a MBW complex with NtMYB6 to regulate the expression of *NtDFR* directly in Chinese Narcissus.

## Methods

### Plant materials

Chinese narcissus cultivar ‘Jin Zhan Yin Tai’ was used in this experiment. Petals, corona, and basal plates were collected for RNA extraction. Tobacco (*Nicotiana tabacum)* was used in stable transformation. Chinese narcissus cultivar ‘Jin Zhan Yin Tai’ and seeds of tobacco were obtained from Institute of Genetics and Breeding in Horticultural Plants, Fujian Agriculture and Forestry University.

### Gene cloning and sequence analysis

Total RNA was extracted from basal plates using Up Plus RNA Kt (TransGen Biotech, Beijing, China). First strand cDNA was synthesized using the PrimeScript™ RT reagent Kit with gDNA Eraser (Perfect Real Time) (Takara, BeiJing, China). The sequence of primer with OligdT is CCAGTGAGCAGAGTGACGAGGACTCGAGCTCAAGCTTTTTTTTTTTTTTTTT. To isolate the full-length open reading frame (ORF) of *NtbHLH* and *NtMYB* genes, 3´ RACE was performed. Specific primers used in three rounds of 3´ RACE PCR amplifications are listed in Supplemental Table 1. After getting the 3´ end, the full-length ORF was amplified by RT-PCR from cDNA using gene-specifc forward and reverse primers (Supplemental Table 1). The total 25 µL PCR mixture (Yesen, Shanghai, China) contained 12.5 µL 2 × Canace® PCR buffer, 1 µL cDNA, 1.25 µL each primer and 8.75 µL double distilled water. The PCR reaction was carried out as follows: pre-heating at 98 ℃for 3 min, 34 cycles at 98 ℃ for 30 s, the annealing temperature was 60 ~ 65 ℃ for 1 min and 72 ℃ for 30 s, then an extension at 72 ℃ for 5 min.

The amino acid sequence of *NtbHLH1* and *NtMYB6* were aligned by using DNAMAN6.0 software with default parameters (Lynnon Corporation, San Ramon, CA, USA). The whole protein sequence of flavonoid-related bHLHs belonging to the subgroup IIIf transcriptional factors and flavnoid-related R2R3-MYBs were used to develop a phylogenetic tree using MEGA7 software with default parameters. Bootstrap was 1000.

### Real time qPCR analysis

The expression of *NtbHLH1* and *NtMYB6* in different tissues of Chinese narcissus was analyzed by real time qRT-PCR using the Light cycler® 480 real time PCR (Roche Diagnostics, Indianapolis, IN, USA) and SYBR Premix Ex Taq (Takara, Beijing, China) according to the manufacturer’s instructions. The conditions of qRT-PCR were as follows: one cycle of denaturation (95 C, 30 s), then 40 amplification cycles (95 ℃, 10 s, 60 ℃, 30 s), and a signal acquisition (72℃, 30 s). The relative expression level was estimated with the Ntactin gene used as an internal standard (Supplementary Table 1). The comparative Ct method was carried out to estimate the gene expression level. Three technical and biological replications for each sample were performed.

### Vector construction and tobacco transformation

Plant expression vector was constructed using the In-Fusion HD Cloing Kit (Takara, Beijing, China). The forward primer was added with an *EcoR*I restriction site and reverse primer with a *Hind*III restriction site (Supplemental Table 1). The sequence was then cloned into expression vector pSAK277. The constructed expression vector pSAK277-NtbHLH1 and pSAK277-NtMYB6 were transferred into *Agrobacterium* GV3101 by freeze–thaw method.

Stable transformation of tobacco used the the leaf disc method [18]. Transformed tobacco shoots were screened on MS basal medium adding 100 mg·L^−1^ Kan and then were confirmed by RT-PCR. The transgenic tobacco plants were then transferred to a mixture of soil for their adaptation under normal condition.

### Total anthocyanin extraction and quantification

The samples were collected from the center of the fresh flowers of transgenic tobacco. Samples were powdered in liquid nitrogen and transferred into a clean tube containing 10 ml of methanol (1% HCl) and kept overnight at room temperature. The extracts were homogenized and centrifuged at 13,000 rpm (10 min, 4 °C) and the upper aqueous phase was subjected to spectrophotometric quantification at 530, 620 and 650 nm using a Spectrophotometer. The relative anthocyanin content was determined using the following formula: OD = (A530 − A620) − 0.1(A650 − A620). The total anthocyanin concentration was determined using a molar extinction coefficient of 46,200 mol·cm^−1^ [40].

### Yeast two hybrid assay

For Y2H experiments, pGADT7 (harboring the GAL4 activation domain (AD)) and pGBKT7 (harboring the GAL4 DNA-binding domain (BD)) were uesd. The ORF fragments of *NtMYB6* and *NtbHLH1* were inserted into pGADT7 and pGBKT7, seperately, generating AD-NtMYB6 and BD-NtbHLH1 construct. The BD-NtbHLH1 was transformed alone or together with AD-NtMYB6 into the yeast strain Y2HGold using the PEG/LiAc method. The autoactivation activities of *NtbHLH1* were tested. The co-transformed colonies were selected on SD medium lacking leucine and tryptophan (SD/-Leu/-Trp), and screened for growth on quadruple dropout SD medium lacking adenine, histidine, leucine and tryptophan (SD/-Ade/-His/-Leu/-Trp). To further confirm the positive interactions, X-α-Gal was used for the assay of β-galactosidase activity.

### Dual luciferase assay

The promoter sequences of Chinese narcissus LAR gene (NtLAR) [31] and DFR gene (NtDFR) [19] were cloned and inserted into multiple cloning site of vector pGreenII 0800-LUC separately as the reporter cassette. pSAK277-*NtbHLH1* and pSAK277-*NtMYB6* driven by the cauliflower mosaic virus (CaMV) 35S promoter were used as the effector cassettes. PSAK277 with β-glucuronidase (GUS) gene was used as the negative control. Dual luciferase assay was conducted in *Nicotiana benthamiana* leaves according to the previous report [4, 11]. The confluent bacteria was resuspended in infiltration buffer (10 mM MgCl2, 10 mM MES, 200 µM Acetosyringone) and incubated at room temperature without shaking for 3 h before infiltration. Firefly luciferase (LUC) values relative to the Renilla luciferase (REN) control were measured using the machine Multifunctional Microplate Reader (Infinite M200 PRO, TECAN, Austria).

## Supplementary Information

**Additional file 1.**

## Data Availability

The sequence of *NtbHLH1* has been deposited in GenBank of NCBI with accession No. of QDS02912.1 (https://www.ncbi.nlm.nih.gov/protein/QDS02912). The sequence of *NtbMYB6* has been deposited in GenBank of NCBI with accession No. of KY645961.1 (https://www.ncbi.nlm.nih.gov/nuccore/KY645961). All other data generated or analyzed during this study are included in this published article.

## Abbreviations

PAs: Anthocyanins and proanthocyanidins
MBW: MYB-bHLH-WDR
Y2H: Yeast two-hybrid
ORF: Open reading frame
RT-PCR: Real-time PCR
qRT-PCR: Quantitative real-time PCR
WT: Wild type
GUS: β-Glucuronidase
TFs: Transcription factors
