# Supplementary material for: NtbHLH1, a JAF13-like bHLH, interacts with NtMYB6 to enhance proanthocyanidin accumulation in Chinese Narcissus
Source: BMC Plant Biol. 2021 Jun 16;21:275. doi: 10.1186/s12870-021-03050-1 (PMC8207774; doi:10.1186/s12870-021-03050-1)
Supplement: Supplementary file 1 — Additional file 1. [file 12870_2021_3050_MOESM1_ESM.docx]

**JAF-like bHLH, *NtbHLH1* Interacts with *NtMYB6* to Enhance Proanthocyanin Accumulation in Chinese Narcissus**

**Yuxin Fan^#^, Jiayu Peng^#^, Jiacheng Wu, Ping Zhou, Ruijie He, Lihui Zeng***

Institute of Genetics and Breeding in Horticultural Plants and College of Horticulture, Fujian Agriculture and Forestry University, Fuzhou 350002, China

*** Correspondence:** Lihui Zeng

lhzeng@fafu.edu.cn

**^#^** these authors contributed equally to this work

Supplementary Material

**Supplementary Table 1. Primers used in this study**

| Primer names Sequences(3´-5´) Description |
| --- |
| NtbHLH1-1F CCAGTGAGCAGAGTGACG cloning 3´ end of *NtbHLH1*  NtbHLH1-1R CTCCTGATGAAGAAACCGAGTTAACC  NtbHLH1-2F GAGGACTCGAGCTCAAGC  NtbHLH1-2R GGTGGAAGGATAAAGAGCAATGTC  NtbHLH1-3F GAGGACTCGAGCTCAAGC  NtbHLH1-3R GAAACTCACTACCAAAGGACTCTCTC  NtMYB6-1F AAAGTGGGGCTTCATAGAGG cloning 3´ end of *NtbMYB6*  NtMYB6-1R CTAATACGACTCACTATAGGGCAAGCAGTGGTATCAACGCAGAGT  NtMYB6-2F AGGAAACATTGGACCAGAAG  NtMYB6-2R CTAATACGACTCACTATAGGGCAAGCAGTGGTATCAACGCAGAGT  NtMYB6-3F ATTCTTTGTTAGGCAACCGATG  NtMYB6-3R CTAATACGACTCACTATAGGGCAAGCAGTGGTATCAACGCAGAGT  NtbHLH1-4F TGGATCCAAAGAATTCATGGCTCAAATTCAAGATGGAATGCAAG pSAK277-NtbHLH1 construction  NtbHLH1-4R TACTCTCGAGAAGCTTTCAGCATTTACCAACAACTCTCTGAAG  NtMYB6-4F TGGATCCAAAGAATTCTTCTACACAAATTAAAATGGGAAG pSAK277- NtMYB6 construstion  NtMYB6-4R TACTCTCGAGAAGCTTCATCATGCGAAGACTGACTCG  NtbHLH1-5F CCGTCAGCTTCATTATCTCCCG qRT-PCR analysis in Chinese narcissus  NtbHLH1-5R GCATTGCTCACCCACGTACACT  NtMYB6-5F TCACACGAACCATCAGCCAAAG  NtMYB6-5R ACAATTCATAGTTCCAACTGCCTG  NtActin-F TGCCCAGAAGTGCTATTCCAG  NtActin-R GTTGACCCACCACTAAGAACAATG  DFR-F AACCAACAGTCAGGGGAATG qRT-PCR analysis in *Nicotiana tabacum*  DFR-R TTGGGCATCGAGAGTTCCAG  ANS-F TGGCGTTGAAGCTCATACTG  ANS-R GGAATTAGGCACACACTTTGC  F3H-F ACAGGGTGAAGTGGTCCAAG  F3H-R CCTTGGTTAAGGCCTCCTTC  F3’H-F TCCAAGAATACTGGCCCAAG  F3’H-R CTCACAACTCTCGGATGCAA  FLS-F GTCCACAACGTTGCATGGTG  FLS-R CACAACTTCTCGCAGCCTC  LAR-F TCAAGGTCCTTTACGCCATC  LAR-R ACGAACCTGCTTCTCTTTGG  ANR-F CATTTGACTTTCCCAAACGC  ANR-R ATTGGGCTTTTGAGTTGTGC  CHS-F GTACAACTAGTGGTGTAGACA  CHS-R CCAACTTCACGAAGGTGAC  UFGT-F CAATGTTTGGGATGGTGTCA  UFGT-R TTCCTCCTCTGCCTCTTTCA  *ACTIN2-F* AATGATCGGAATGGAAGCTG  *ACTIN2-R* TGGTACCACCACTGAGGACA  ADMYB6-F GGAGGCCAGTGAATTCTTCTACACAAATTAAAATGGGAAG pGADT7- NtMYB6 construction  ADMYB6-R CGAGCTCGATGGATCCCATCATGCGAAGACTGACTCG  BDbHLH1-F CATGGAGGCCGAATTCATGGCTCAAATTCAAGATGGAATG pGBKT7- NtbHLH1 construction  BDbHLH1-R GCAGGTCGACGGATCCTCAGCATTTACCAACAACTCTCTG |


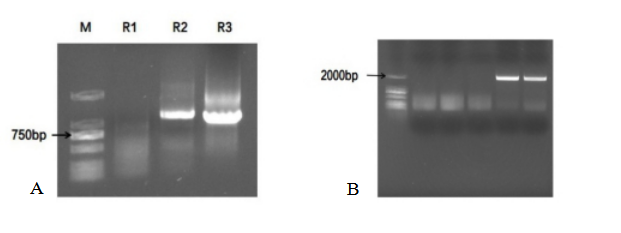


## Supplemental Figure 1. 3’RACR amplified product of *NtbHLH1* ( R1：first round；R2：second round；R3：third round；M：Marker ) ( A ) ; RT-PCR amplification of *NtbHLH1* ORF from Chinese narcissus ( B ).

##
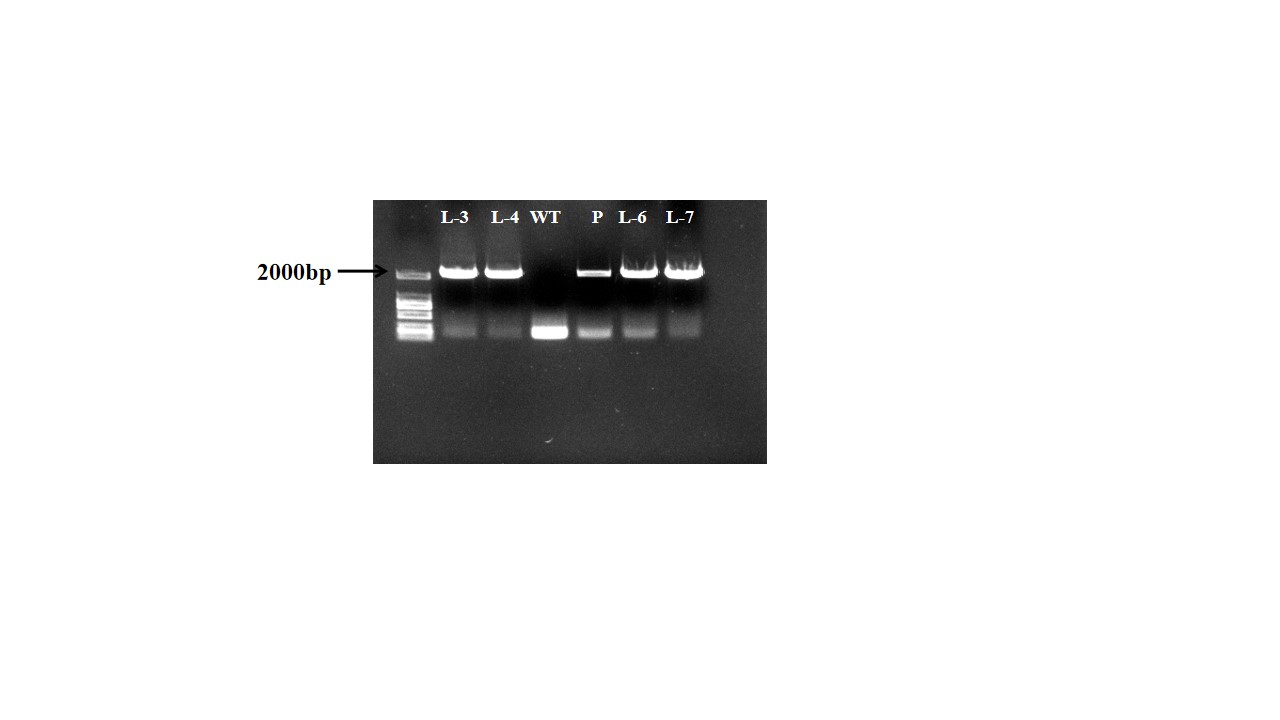
Supplemental Figure 2. The PCR analysis of trangenic tobacco plants of *NtbHLH1*. Four transgenic lines (L-3, L-4, L-6 and L-7) . wild type (WT) and pSAK277-NtbHLH1 ( P ).

##
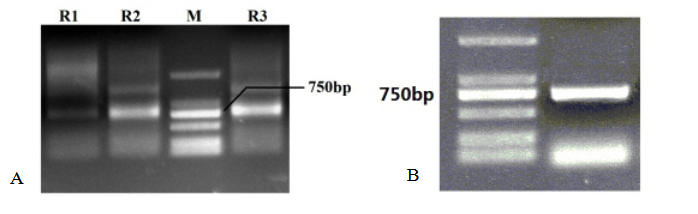


## Supplemental Figure 3. 3’RACR amplified product of *NtbMYB6* ( R1：first round；R2：second round；R3：third round；M：Marker ) ( A ) ; RT-PCR amplification of *NtbMYB6* ORF from Chinese narcissus ( B ).

##
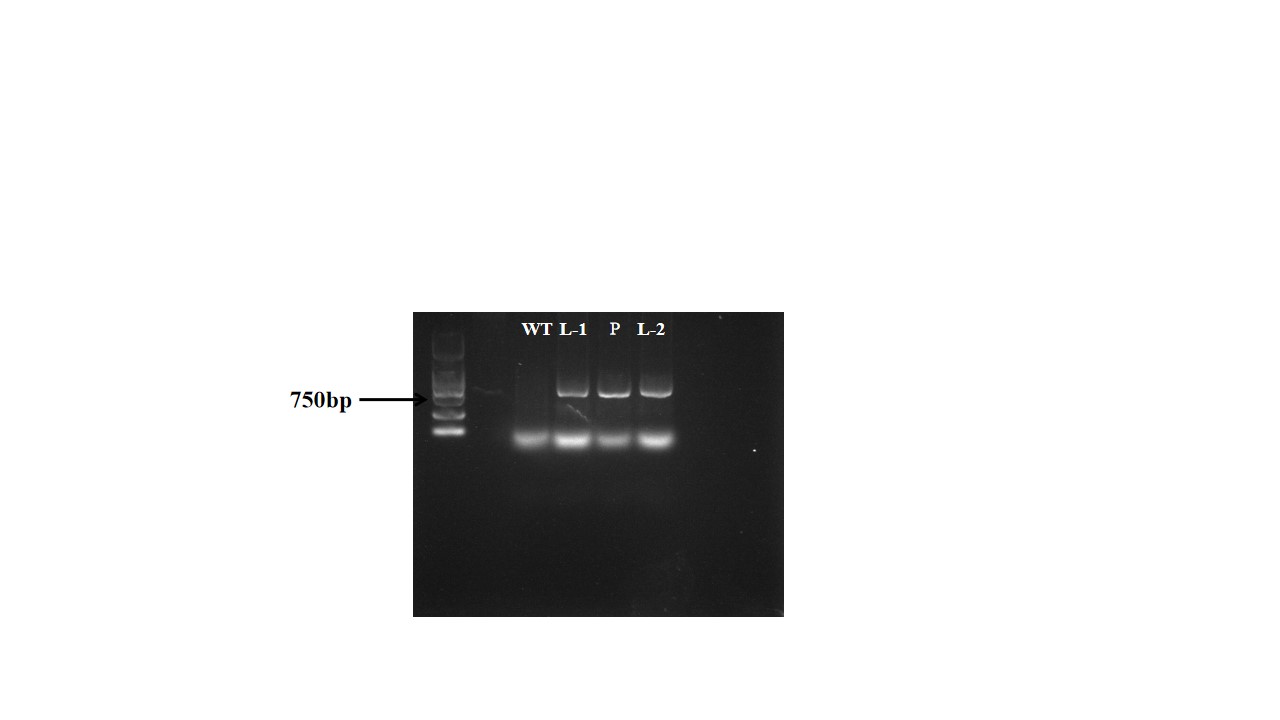


## Supplemental Figure 4. The PCR analysis of trangenic tobacco plants of *NtbMYB6*. Four transgenic lines (L-1 and L-2) . wild type (WT) and pSAK277-NtMYB6 ( P ).
